# Effectiveness of the “Create Sensitivity” Caring Model on Blood Glucose/ Glycosylated Hemoglobin and Quality of Life in Patients With Type 2 Diabetes

**DOI:** 10.2174/1874434601812010195

**Published:** 2018-09-18

**Authors:** Davood Hekmatpou, Ali Poorgharahkhan, Mahbobeh Sajjadi, Amir Javaheri

**Affiliations:** 1 Nursing. Faculty member of Arak University of Medical Sciences, Arak, Iran; 2 Master of Science in Nursing. Arak University of Medical Sciences,. Arak, Iran; 3 Faculty member of Arak University of Medical Sciences, Arak, Iran; 4 Endocrinologist. Kurdistan University of Medical Sciences. Kurdistan, Iran

**Keywords:** Blood glucose, Glycosylated hemoglobin, “Create Sensitivity” caring model, Quality of life, Type 2 diabetes, Endocrine disorder

## Abstract

**Objective:**

This study aimed to determine the effectiveness of the “Create Sensitivity” caring model on blood glucose/ glycosylated hemoglobin and quality of life in patients with type 2 Diabetes.

**Methods:**

This study enrolled 70 patients from an educational hospital in Kurdistan, Iran. The model was implemented among the test group over a period of 3 months. Blood glucose/ glycosylated hemoglobin and patients’ quality of life were measured before and after intervention. Data were analyzed using version 21 of the statistical software SPSS.

**Results:**

After the intervention, significant differences existed between the test and control groups both in blood glucose levels (means, 146.4 ± 51.3 mg/dl and 175.6 ± 59.8 mg/dl, respectively; *P*=0.032) and in glycosylated hemoglobin (means, 67.89 ± 13.34 mmol/mol and 80.03 ± 17.234 mmol/mol, respectively; *P*= 0.002). Additionally, there was also a significant difference between the quality of life of the patients in test group (mean, 58.25 ± 5.3) and that in the control group (mean, 47.02 ± 4.5) (*P*= 0.0001).

**Conclusion:**

Use of this model was associated with reducing fasting blood glucose and glycosylated hemoglobin and increasing the total mean of quality of life in the patients in the test group. So, the application of this model is recommended.

## 1 INTRODUCTION

Diabetes mellitus is the most common endocrine disorder and one of the most important health challenges to emerge in the 20th century due to sedentary lifestyles leading to more individuals acquiring diabetes or pre-diabetes (otherwise known as the metabolic syndrome) in middle age or even earlier [1]. In 2012, the number of patients with diabetes worldwide was about 371 million [2], of which 70% lived in developing countries [3]. Four million people in Iran had diabetes as of 2010 [4]. Recent studies have shown that 7.8% of the 25-56 year-old population has diabetes [4]. At present, the disease is the fifth-leading cause of death in Western societies, and the fourth most-common cause of a visit to the physician [5]. Type 2 diabetes, as the most common type of diabetes, includes 90% of all cases of diabetes [6].

Rapid economic development and urbanization have led to an increase in the number of patients with diabetes. Other factors in the development of this disease are changes in lifestyle that reduce physical activity; an increase in the consumption of refined carbohydrates; and obesity, aging of the population, and decreasing quality of life [7]. The diverse and relatively common complications of diabetes are retinopathy, nephropathy, neuropathy [8], and peripheral arterial diseases [9]. These complications cause a heavy economic burden and reduce patients’ quality of life [10]. The ultimate objective in the treatment of diabetes is glycemic control in the normal range without hypoglycemia [6]. One of the biggest challenges for these patients is learning how to live with diabetes, control blood sugar complications, and promote their quality of life [11]. To accomplish this aim, we selected the “Create Sensitivity” caring model, earlier developed and described by Hekmatpou *et al.* to control the symptoms of chronic diseases such as congestive heart failure [12]. This model was built based on the fundamental assumption that not only is the lack of awareness of patients with respect to the factors that lead to hospital readmission for a chronic problem (and one that also often leads to readmission), but also that a targeted strategy to address this could be an effective mechanism to control acute complications of chronic diseases (such as diabetes). So, this study aimed to determine the effectiveness of this concept through the implementation of the “Create Sensitivity” caring model in patients with type 2 Diabetes for the purpose of controlling blood glucose / glycosylated hemoglobin and improving quality of life.

## 2 METHODS

This is a quazi-experimental study. Using the “Create Sensitivity” caring model was an independent variable and blood glucose, glycosylated hemoglobin, and quality of life were three dependent variables of this study. This interventional study included all patients with type 2 diabetes who were referred to the diabetes clinic and/or the emergency unit of educational hospital in Kurdistan, Iran.

The sample size was determined *via* use of sample size equation and factors (d=1.1, S_1=_ 1.74_,_ S_2=_ 1.35, β= 0.2, and α= 0.05) [13]. The sample size was determined to be 64 patients in both groups. Based on the nature of interventional studies, it is considered that 5% to 10% of the study population would be lost to attrition [13]. So, 70 patients with type 2 diabetes were initially selected to take part in the study. After obtaining informed consent, the patients were randomly assigned into 2 groups of equal size (intervention, n=35; control, n= 35). Inclusion criteria were: confirmed type 2 diabetes, the ages between 30 and 60 years, at least 4 months after the original diagnosis of diabetes and taking medications, and blood glucose above 200 mg/dl at baseline, and absence of either mental health problems or other incurable diseases (according to each patient’s statements). Exclusion criteria included migration, lack of cooperation/being lost to follow-up, and the patient’s death.

Patients were studied in 2 groups of 35 subjects. The test group comprised of 17 men (48.6%) and 18 women (51.4%), and the control group 18 men (51.4%) and 17 women (48.6%). In the test group, the mean age was 50.6 ± 7.8 years and the mean BMI was 26.7 ± 3.6 years. In the control group, the mean age was 50.4 ± 7.3 years and the mean BMI was 26.8 ± 2.8 years. About 29% of participants were under 40 and 71% over 40 years.

### 2.1 Intervention

The “Create Sensitivity” caring model Fig. (**1**) consists of four administrative stages that include cognitive reconstruction (during which patients and their families take part with the doctors and nurses comprising the patient’s healthcare team), a comprehensive and systematic patient discharge program, scientific self-treatment, and the development and maintenance of hope among patients. Two of these 4 stages were implemented in health centers, while the other 2 were followed at each patient’s home for 3 months after the intervention. The model was based on a general policy or strategy of building sensitivity (or creating sensitivity) among the medical team, the patients, and their families. The stages of the model were implemented as an overall strategy of care in the intervention group. Further details on this strategy appear in the next sections.

#### 2.1.1 Pre Intervention

At first, after coordination with officials of the city health network, all necessary measures, such as assigning of the seminar hall, the patient’s education classes, and an office for follow-up, were provided to implement the model. Then, during a meeting with officials of the various health departments, it was announced to all related units to record the reasons with the relevant authorities for the readmission of patients due to lack of glycemic control. This coordinated effort was a factor that built awareness among the treatment team with respect to the reasons for repeated visits/readmissions due to lack of glycemic control.

#### 2.2.2 Intervention

##### 2.2.2.1 Cognitive Reconstruction

A) *Cognitive reconstruction of the treatment team:* A two-hour conference (including host speakers and discussion) about the causes and risk factors of type 2 diabetes readmission was conducted with the purpose of creating and reinforcing awareness of these factors among the medical team. The participants were asked to record the reasons for their repeated readmissions in their nursing reports, and inform their physicians so that they also become sensitive to the issue.

- *Cognitive reconstruction in patients and their family members:* Face-to-face educational classes were held in a small group of patients it covered the factors of readmission and repeated visits, lack of control of blood sugar, complications and consequences of the disease, and self-care for patients and their families, as well as how to control the disease through a comprehensive regimen including diet, medication, and proper life style choices. Some of these education classes were held outside the main site (the hall) due to elderly patients being enrolled. Therefore, education booklets were used along with an educational CD for the implementation of the “Create Sensitivity” program in patients. They received individual instruction and more multimedia cases for private use at home and prevention of forgetfulness. Also, regular visits were announced by physicians of the research team to patients and their families. The patients were to return for follow-up and examination on a specific date.

##### 2.2.2.2 A Comprehensive Program of Systematic Patient’s Discharge

Two nurses with clinical experience relevant to the treatment of diabetes were selected as assistants of the research team. They were responsible for implementing programs in health centers and follow-up of patients at home under the supervision of a primary researcher. During these programs, they trained patients with respect to self-care skills and provided supplementary educational materials. Then, comprehensive patient-discharge program was designed. Before discharge, a one-hour recall session was held to help patients remember the following self-care and collaborative (with the healthcare team) treatment techniques (in addition to the training given):

- How to prepare and follow a diabetic diet, adhere to the medication regimen, and implement lifestyle changes (including increases in physical activity).

- How to report progress and collaborate with two nurses after discharge. In this regard, CDs, pamphlets, videos, and photos were provided. Two patients with diabetes who had successfully controlled their blood sugar were also invited to take part in the class in order to relay their experiences to the other study enrollees.

##### 2.2.2.3 Scientific Self-Treatment

To achieve “scientific self-treatment”, which for the purposes of this paper is defined as the building of knowledge and techniques that allow the translation of knowledge related to diabetes into independent action to control the disease, the following interventions were performed.

A) *Continuous follow-up:* Patients were asked to communicate with the research team in person and by telephone to obtain solutions if they encountered any problems or had questions.

B) *Creating incentives:* To motivate and sustain adherence to a medical regimen, the research team sent a short message each week (*via* SMS) or contacted them *via* telephone. The team reminded each patients of the training checklist, which contained many of the tips that had previously been discussed.

C) *Identifying high-risk situations and aiding with compliance:* Patients were given self-control strategies to balance their lifestyles.

D) *Increasing self-efficacy:* This intervention aimed to eliminate the top-down nurse – patient relationship in favor of the patient playing the role of “associate” in their healthcare and thereby monitoring his or her own behavior. Self-efficacy in this context referred to patients accepting responsibility and increasing their activity of daily living.

*E) Removal of misconceptions:* This intervention aimed to modify wrong beliefs about some behaviors. For example some patients have expected that taking opioids can decrease their blood glucose.

F) *The management of relapse:* One week after discharge, the patients were invited (either by phone or in person) to return as a group to discuss misperceptions and recall their training. The correct solutions to these types of challenges were also re-taught during this session, with the aim of strengthening patients’ sense of responsibility, potential benefits, and sense of being a part of the care system.

##### 2.2.2.4 Development of Hope

- *Giving hope:* Through frequent calls with patients using phone, or mobile and SMS, particularly through the use of short messages.
- Advice and encouragement to continue treatment and participation in social occasions and activities that makes them happy.
- Introducing specific patients for psychiatric consultation.
- *Positive role models*: Introducing and connecting patients with people who have successfully controlled diabetes in their own lives.
- *Self-management of diabetes:* Training was provided to address the following important items:
  1. Patients’ self-management with respect to adjusting their wishes and objectives.
  2. Encouragement was provided with respect to helping patients control their motivations and abandon poor habits.
  3. Tendency management (for example, noting that a given habit, such as self-control, is off track).
  4. Understanding the mental path that leads to recurrence and relapse.

### 2.2 Data Gathering Tests

All fasting blood samples of both groups were tested using the PARS AZMOON Kit (Pars Azmoon, Tehran, Iran) (normal range, 70-110 mg/dl). HbA1C testing was performed using a NycoCard kit (Alere; Waltham, Massachusetts, USA) (normal range, 4.5% -6.3%).

The Diabetes Quality-of-Life Measure (DQOL) was used for quality of life measurement; it was used for the first time in Iran by Nasyhatkon *et al.* (r=0.72, α=0.77). The DQOL is a Likert-type questionnaire in which 15 questions about 6 quality of life dimensions are posed, with “scores ranging from at least 15 to 75” [14].

All data were analyzed using SPSS 21 (X^2^, paired t-test and t-test) (IBM; Armonk, New York, USA); no patients were lost to follow-up.

### 2.3 Ethical Considerations

This study was approved by the Medical Ethics Committee of Arak University of Medical Sciences with the code: “MUK.REC.1394 (2015).49”. Each participant was recruited/ enrolled in the study after filling out the consent form.

## 3 RESULTS

The results of this study were measured after 3 months against the baseline measurements with respect to fasting blood sugar, glycosylated hemoglobin, and BMI. Importantly, the test and control groups were homogenous and without statistically significant differences based on chi-square test, Fisher's exact test and independent t test by demographic characteristics (sex, level of education, occupation, and age). Analysis of variance showed no significant difference in the three measurements that were compared at 3 months versus baseline based on any of these demographic characteristics. After 3 months of training according to the tenets of the “Create Sensitivity” model, mean of fasting blood glucose and glycosylated hemoglobin (laboratory parameters) in both groups had improved. The results of independent t-tests showed that the differences in the means for both fasting blood glucose and glycosylated hemoglobin were statistically significant between the two groups, and the paired t-test results suggest that, in the test and control groups, the difference in means for fasting blood glucose and glycosylated hemoglobin were significant before and after training Tables **1** and **2**.

The mean quality of life (as measured by the DQOL) in this study was at an intermediate level prior to the intervention (47.31 ± 5.30). The mean quality of life in patients in the test group increased post-intervention. A significant difference existed between the total mean of quality of life of the patients in the test group (58.25 ± 5.3) with the total mean of quality of life of the patients in control group (47.02 ± 4.5) (*P*=0.0001) Table **3**.

The post-intervention results show that quality of life measures had improved significantly in the test group for dimensions such as treatment satisfaction, emotions, and satisfaction with socio-personal relationships (*P* ≤ 0.0001). Furthermore, after the intervention, the dimensions of diabetes-related anxiety and personal limitations were decreased; this was also statistically significant (*P*≤ 0.0001). The dimension of social concerns (job) was about the same as before and after the intervention. Overall, these results showed that the implementation of this model was effective in improving the quality of life among the recruited patient population Tables **4**, **5**.

## 4 DISCUSSION

The main goal of diabetes treatment is to achieve an appropriate and acceptable level of blood glucose, which is associated with the reduction of morbidity and mortality. The findings of this study showed that fasting blood glucose and glycosylated hemoglobin levels significantly decreased in both groups due to medication usage, but the reduction of fasting blood glucose in the test group was 32.7% versus 15% in the control group. Similarly, levels of glycosylated hemoglobin were reduced by 18% in the test group versus 6.5% in the control group. These differences may be related to the implementation of the “Create Sensitivity” caring model for the medical team, patients with diabetes, and their families.

The results of this study are similar to results reported in other studies on the implementation of caring models. Mohammad-Zadeh *et al.* studied 32 patients with type 2 diabetes that were enrolled in an intervention program devised by a diabetes association in Iran. Before exercise training, the importance and impact of exercise on the control of type 2 diabetes were described in a speaking session with patients who were also given an educational illustrated book that included information on how to exercise (with diagrams/examples), different types of movement to perform and the recommended exercise sequence. Based on the model, 3 months after training, a significant decrease was observed in the frequency of abnormal fasting blood sugar (15.6%) and glycosylated hemoglobin (28.1%) compared with before the intervention [14].

In a study entitled “Making Sensitivity” caring model in patients suffering from congestive heart failure” by Hekmatpou *et al.*, (2009), it was concluded that this model had a positive effect on most of the parameters related to control of congestive heart failure, including mean re-admission, BMI, blood indices, and the activities of daily living. A significant difference was also seen between the two groups (the intervention and control) in these variables [15].

Although the effects of the “Create Sensitivity” caring model have not been studied in patients with diabetes, the impact of different models has been evaluated on glycemic control in diabetic patients [13, 16].

In a study on the effect of the training program based on the health belief model on reducing the level of glycosylated hemoglobin in patients with type 2 diabetes, 138 patients in the diabetes center at Zahedan (a city in South East of Iran) demonstrated a significant decrease in the level of glycosylated hemoglobin in the intervention group (from 9.63 mg/dl before the intervention to 8.30 mg/dl 3 months after the intervention) [16].

With respect to investigating the effect of patient education on blood glucose control in patients with type 2 diabetes based on Basnef’s model, a study was conducted in 100 patients with type 2 diabetes in a clinic in Shiraz (a city in the south of Iran). The level of glycosylated hemoglobin after 3 months of educational intervention in the test group declined from 8.65 mg/dl before intervention to 7.47 mg/dl after intervention, and in the control group from 8.57 mg/dl to 8.51 mg/dl. Fasting blood glucose in the intervention group was from 207.1 mg/dl to 124.2 mg/dl and in the control group from 181 mg/dl to 186.3 mg/dl [13].

Another study was carried out in 60 patients with type 2 diabetes in Mashhad, Iran to examine the effect of a care program based on Roy’s model of psychological adjustment. In this study, an educational intervention was performed for 4 months. The level of glycosylated hemoglobin in the test group declined from 9.14 mg/dl to 7 mg/dl before and after intervention, respectively; in the control group, this decline was from 9 mg/dl to 8.6 mg/dl [17].

In the present study, the increased scores for all dimensions of quality of life after intervention in the test group showed that the “Making Sensitivity” model could lead to changes in attitudes, knowledge, and practice levels among patients with type 2 Diabetes and promote improved quality of life. These results are similar to other related studies performed in Iran. In their study on quality of life in patients with type 2 diabetes, Taghdisi *et al.* have stated that the mean scores of psychological and physiological health in patients before and after interventions statistically significantly increased in the test group [18]. Ghotbi *et al.* showed that using a model could increase self-care behaviors in a test group after intervention and that this could improve quality of life [19]. Zare Bahramabadi *et al.* [20] reported that mean scores for general health, role-playing deficit limitation due to emotional issues, social functioning, vitality, psychological health, body functions/role-playing deficit limitation due to body functions, and somatic pain all improved significantly with the use of cognitive behavioral therapy [20]. Comparison of the results of this cross-section of studies with this study showed that the “Create Sensitivity” model can be a practical caring model for providing care to all patients with type 2 Diabetes due to its simplicity, applicability, and efficiency.

## CONCLUSION

Our findings showed that the use of “Create Sensitivity” caring model was associated with reducing fasting blood glucose and glycosylated hemoglobin in patients with type 2 diabetes down below the upper border that constitutes control levels. Additionally, the quality of life scores were increased so that the test group demonstrated what could be considered a good quality of life. Given the increase in the number of individual with diabetes, finding ways to change the habits and improve the quality of life in these patients is important. Therefore, educational programs, which are clinically beneficial to patients, should be used to help control type 2 diabetes [21]. These programs should be practicable according to the culture and traditions of the community. It is suggested that the “Create Sensitivity” caring model could be applied to those with diabetes due to the chronic nature of the disease, the treatment costs, the irreparable effects of diabetes, and the efficiency of the “Create Sensitivity” caring model to control blood glucose and the complications of the disease.

One of the limitations of this study was the short time period of the intervention. It is likely that an intervention lasting at least 6 months would lead to better control of blood glucose and glycosylated hemoglobin in patients with type 2 Diabetes.

## Figures and Tables

**Fig. (1):**
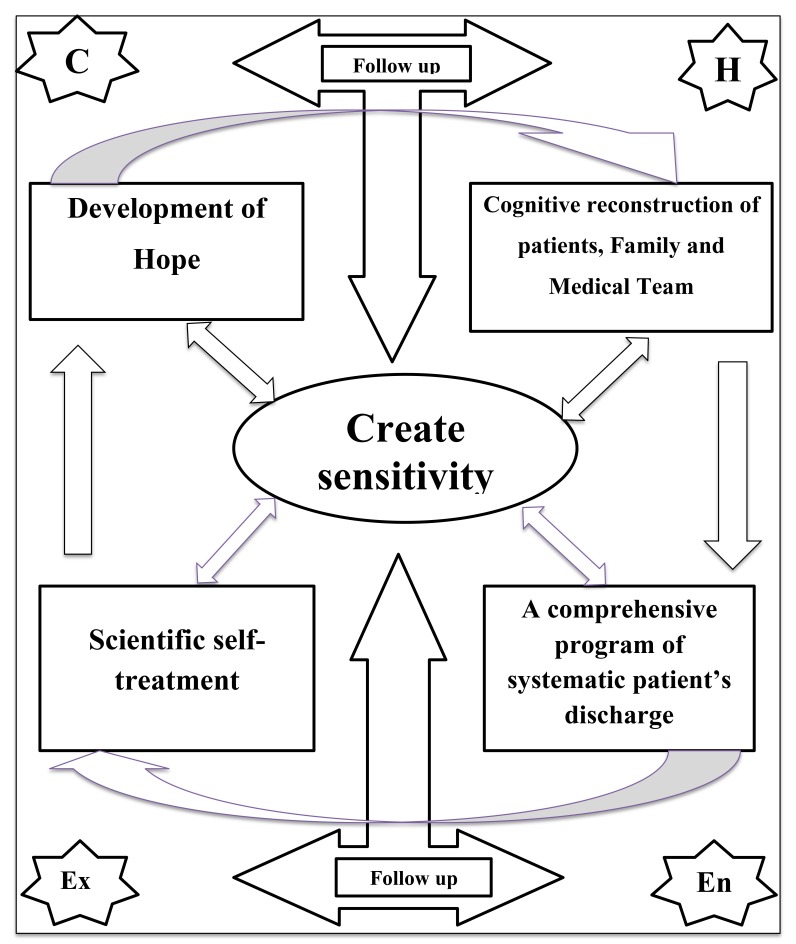
"Create Sensitivity” caring model. (C: Community, H: Hospital, En: Entrance to hospital, Ex: Exit to community).

**Table 1:** Demographic data of two test and control groups in the study of implementation of “Create Sensitivity” caring model

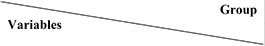	**Control Group**		**Test Group**		Group Variables	
	%	Frequency	%	Frequency		
*p*-value=0.811	48.6	17	51.4	18	Female	**Sex**
	51.4	18	48.6	17	Male	
Fisher exact=2.986 *p*-value=0.781	14.3	5	7.5	2	Illiterate	**Education**
	40	14	1.37	13	Primary education	
	14.3	5	20	7	Secondary education	
	28.6	10	28.6	10	Diploma	
	0	0	2.9	1	Technician	
	2.9	1	5.7	2	Up graduate	
Fisher exact=3.706 *p*-value=0.637	40	14	42.9	15	House Wife	**Job**
	31.4	11	17.1	6	Business	
	14.3	5	22.9	8	Clerk	
	8.6	3	11.4	4	Retired	
	2.9	1	0	0	Worker	
	2.9	1	5.7	2	Farmer	
t- test *p*-value=0.743	17.1	6	14.3	5	>40	**Age/year**
	82.9	29	85.7	30	<40	

**Table 2:** Comparing of the means of Fasting Blood Sugar between two test and control groups in the study of implementation of “Create Sensitivity” caring model.

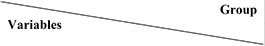	**Test Group**	**Control Group**	* **Pv**
**FBS(Before)**	105.5 mg/dl ±217.7	90.5 mg/dl ±206.7	0.642
**FBS(After)**	51.3 mg/dl ±146.4	59.8mg/dl±175.6	0.032
** pv	0.0001	0.04	

* t- test ** Paired t-test

**Table 3:** Comparing of the means of HBA1C between two test and control groups in the study of implementation of “Create Sensitivity” caring model.

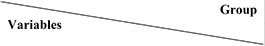	**Test Group**		**Control Group**		* **Pv**
	%	mmol/mol	%	mmol/mol	
70.84	20.72±86.31	1.9±10.05	22.97±87	2.12±10.12	**HBA1C(Before)**
**HBA1C(After)**	1.2±8.3	13.34±67.89	1.5±9.4	17.21±80.03	0.002
** pv	0.0001		0.018		

* t- test ** Paired t-test

**Table 4:** Comparing of the total means of Quality of life of the patients between two test and control groups in the study of implementation of “Create Sensitivity” caring model.

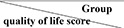	**Test Group**	**Control Group**	***P*-Value/(T-Test)**
**Before**	48.31 ± 5.30	46.48 ± 4.5	0.108
**After**	58.25 ± 5.3	47.02 ± 4.5	0.0001
** (Paired t-test)/** **p-Value**	0.0001	0.113	–

**Table 5:** Comparing of the means of dimensions of Quality of life of the patients between two test and control groups in the study of implementation of “Create Sensitivity” caring model.

**Group** **Dimensions of Quality of Life**	**Time**	**Test Group**	**Control Group**	**T-Tests/ P-Value**
		**M±SD**	**M±SD**	
**Treatment Satisfaction**	Before	2. 9±21.31	2.29±21.08	0.06
	After	2. 9±26. 71	2. 9 ±21.97	0.0001
**P-Value/** **(Paired t-test)**		0.0001	0.07	
**Emotions**	Before	1. 7 ±10.48	2. 38 ±9.2	0.013
	After	1.6 ±11.8	1. 9 ±9.17	0.0001
**P-Value (Paired t-test)**		0.019	0.838	
**Satisfaction with socio- personal relationships **	Before	1.17 ±6.28	1. 4 ±6.05	0.469
	After	1.26 ±7. 57	1.4 ±5.8	0.0001
**P-Value (Paired t-test)**		0.016	0.083	
**Anxiety about diabetes**	Before	1. 38 ±6.71	1. 36 ±6.8	0.795
	After	1. 3 ±4.5	1. 3 ±6.8	0.0001
**P-Value (Paired t-test)**		0.0001	0.521	
**Social concerns - jobs**	Before	1. 41 ±5.9	1. 2 ±5.6	0.292
	After	1.13 ±5.6	1. 17 ±5.54	0.62
**P-Value (Paired t-test)**		0.07	0.487	
**Limitations**	Before	0.97 ±6.40	1. 3 ±6.82	0.131
	After	0. 81 ±4.57	1. 06 ±6.7	0.0001
**P-Value (Paired t-test)**		0.0001	0.499	
